# Patient satisfaction with 6-month paliperidone palmitate versus other long-acting injectable antipsychotics

**DOI:** 10.1192/j.eurpsy.2024.1509

**Published:** 2024-08-27

**Authors:** A. Pérez-Balaguer, I. García del Castillo, B. Gómez-Esteban, M. Vicente-Burguillo, H. Vizcaíno-Herrezuelo, S. Castelao-Almodóvar, A. Arce de la Riva, F. Neira-Serrano

**Affiliations:** ^1^Psychiatry, El Escorial Hospital; ^2^Psychiatry, Puerta de Hierro University Hospital; ^3^Statistics, Francisco de Vitoria University, Madrid, Spain

## Abstract

**Introduction:**

Long-acting injectable antipsychotics (LAIs) offer advantages for schizophrenic patients compared to oral antipsychotics: less frequent dosing, lower relapse rates, better adherence, and lower healthcare costs. LAIs include paliperidone, aripiprazole, olanzapine, risperidone, and zuclopenthixol. Paliperidone palmitate is the only antipsychotic with two formulations with an administration interval longer than one month (3-monthly and 6-monthly), which could be better for the patient and help ensure treatment continuity, especially in cases of limited access to the health care system.

**Objectives:**

To assess the satisfaction of patients under treatment with 6-month paliperidone palmitate compared to other long-acting injectable antipsychotics with a higher frequency of administration.

**Methods:**

We analyzed the satisfaction level of a sample of patients receiving treatment with LAIs at the Mental Health Center of El Escorial. All patients had a diagnosis of schizophrenia or other psychotic disorders (according to DSM-5). Patients who met the inclusion criteria completed the Treatment Satisfaction Questionnaire for Medication (TSQM), a generic questionnaire of treatment satisfaction that measures four dimensions: side effects, treatment efficacy, comfort of use, and overall satisfaction. Other clinical and socio-demographic variables were collected, as well as the type of injectable, dose, and frequency of administration.

**Results:**

Data from approximately 30 patients will be analyzed and discussed later.

**Conclusions:**

Less frequent administration of LAIs may result in greater patient satisfaction and be just as beneficial clinically. Treatment satisfaction is positively associated with an improvement in psychotic symptoms and seems to be related to better adherence.

**Disclosure of Interest:**

None Declared

